# Abortigenic but Not Neurotropic Equine Herpes Virus 1 Modulates the Interferon Antiviral Defense

**DOI:** 10.3389/fcimb.2018.00312

**Published:** 2018-09-12

**Authors:** Katrien C. K. Poelaert, Jolien Van Cleemput, Kathlyn Laval, Herman W. Favoreel, Gisela Soboll Hussey, Roger K. Maes, Hans J. Nauwynck

**Affiliations:** ^1^Department of Virology, Immunology and Parasitology, Faculty of Veterinary Medicine, Ghent University, Merelbeke, Belgium; ^2^Lewis Thomas Laboratory, Department of Molecular Biology, Princeton Neuroscience Institute, Princeton University, Princeton, NJ, United States; ^3^Department of Pathobiology and Diagnostic Investigation, College of Veterinary Medicine, Michigan State University, East Lansing, MI, United States

**Keywords:** equine herpesvirus 1, upper respiratory tract, innate immunity, type I interferon, phenotype

## Abstract

Equine herpesvirus 1 (EHV1) is considered as a major pathogen of *Equidae*, causing symptoms from mild respiratory disease to late-term abortion and neurological disorders. Different EHV1 strains circulating in the field have been characterized to be of abortigenic or neurovirulent phenotype. Both variants replicate in a plaque-wise manner in the epithelium of the upper respiratory tract (URT), where the abortigenic strains induce more prominent viral plaques, compared to the neurovirulent strains. Considering the differences in replication at the URT, we hypothesized that abortigenic strains may show an increased ability to modulate the type I IFN secretion/signaling pathway, compared to strains that display the neurovirulent phenotype. Here, we analyze IFN levels induced by abortigenic and neurovirulent EHV1 using primary respiratory epithelial cells (EREC) and respiratory mucosa *ex vivo* explants. Similar levels of IFNα (~70 U/ml) were detected in explants inoculated with both types of EHV1 strains from 48 to 72 hpi. Second, EREC and mucosa explants were treated with recombinant equine IFNα (rEqIFNα) or Ruxolitinib (Rux), an IFN signaling inhibitor, prior to and during inoculation with abortigenic or neurovirulent EHV1. Replication of both EHV1 variants was suppressed by rEqIFNα. Further, addition of Rux increased replication in a concentration-dependent manner, indicating an IFN-susceptibility for both variants. However, in two out of three horses, at a physiological concentration of 100 U/ml of rEqIFNα, an increase in abortigenic EHV1 replication was observed compared to 10 U/ml of rEqIFNα, which was not observed for the neurovirulent strains. Moreover, in the presence of Rux, the plaque size of the abortigenic variants remained unaltered, whereas the typically smaller viral plaques induced by the neurovirulent variants became larger. Overall, our results demonstrate the importance of IFNα in the control of EHV1 replication in the URT for both abortigenic and neurovirulent variants. In addition, our findings support the speculation that abortigenic variants of EHV1 may have developed anti-IFN mechanisms that appear to be absent or less pronounced in neurovirulent EHV1 strains.

## Introduction

Every 24 h, 100,000 liters of air with potential pathogens pass through the respiratory system of an adult horse. Depending on the size of the pathogen particle, it can penetrate into the upper or lower respiratory tract (Derksen, 1999). A highly prevalent pathogen in horse populations worldwide that causes infection via the respiratory route is equine herpes virus 1 (EHV1). EHV1 is a member of the *Varicellovirus* genus in the *Alphaherpesvirinae* subfamily. The virus spreads via respiratory secretions during direct or indirect contact. EHV1 replicates in the epithelium of the upper respiratory tract (URT), causing mild respiratory problems. Via single infected local immune cells, EHV1 can penetrate through the basement membrane (BM), enter the blood stream and disseminate to the pregnant uterus or the central nervous system. Infected leukocytes may transmit virus to endothelial cells of the endometrial or central nervous system vasculature, resulting in thromboembolic disease and ischemia, causing neonatal foal death, late term abortion, or neurological disorders, such as hind limb ataxia (Edington et al., 1986, 1991; Smith et al., 1993, 2004; Smith, 1997; van der Meulen et al., 2000; Smith and Borchers, 2001; Goehring et al., 2006; Gryspeerdt et al., 2010; Laval et al., 2015). The occurrence of clinical symptoms has extensive economical consequences caused by treatment, biosafety, and quarantine procedures and interruption of training and competition times (Lunn et al., 2009). Many horses become latently infected early in life and remain carriers of the virus (Kydd et al., 1994; Slater et al., 1994).

Different EHV1 strains circulating in the field have been characterized as abortigenic or neurovirulent phenotypes, based on a single nucleotide polymorphism in the catalytic subunit of the viral DNA polymerase (Nugent et al., 2006). Lunn et al. (2009) demonstrated that the majority of equid herpes myeloencephalopathy (EHM) outbreaks are associated with the neurovirulent variants, whereas most abortions involved abortigenic strains. *In vivo* and *ex vivo* studies indicated that both EHV1 phenotypes replicate in a plaque-wise manner in the epithelium of the URT (Gryspeerdt et al., 2010; Vandekerckhove et al., 2010). Abortigenic variants replicate more efficiently in the URT, evidenced by a higher amount of larger viral plaques, whereas the neurovirulent strains infect local immune cells much earlier [24 h post inoculation (hpi)] in infection compared to abortigenic strains (36 hpi). This correlates with a cell-associated viremia earlier in infection, which might favor onset of neurological disease (Gryspeerdt et al., 2010; Vandekerckhove et al., 2010; Laval et al., 2015).

As the initial exposure and primary viral replication occur in the epithelium of the URT, an effective innate immune response to EHV1 at this anatomical site is of key importance (Alberts et al., 2002). An overlying mucoprotein network, epithelial cell layers and their intercellular bridges form the first innate physical barrier, which complicates the attachment and invasion of pathogens into the epithelial cells (Vareille et al., 2011; Yang et al., 2012; Volsko, 2013; Van Cleemput et al., 2017). Moreover, these cells have the ability to secrete peptides, such as lactoferrins, defensins and nitric oxide, which have direct antimicrobial activities (Brandtzaeg, 1997; Oppenheim et al., 2003). Another key player in the host defense against viral infections of the URT is the interferon (IFN) system (Samuel, 2012). IFNs are secreted cytokines, which establish an antiviral state in cells (De Maeyer and De Maeyer-Guignard, 1988, 1998). Three types of IFN (α/β, λ, and γ) can be distinguished and bind to distinctive but related cell surface receptor complexes (de Weerd and Nguyen, 2012). During the first critical hours of a viral infection of respiratory epithelial cells, IFNα, -β (type I IFN), and IFN-λ (type III IFN) are secreted and show similar antiviral activities via a highly similar signal transduction cascade. Type II IFN or IFNγ binds to the IFNGR complex and is a key mediator of virus-specific cellular immunity (Sadler and Williams, 2008). Conserved motifs within classes of potential pathogens are recognized through specific pathogen recognition receptors (PRR), such as toll-like receptors (TLR), expressed by professional immune cells and the majority of non-hematopoietic cells, including epithelial cells (McClure and Massari, 2014). Airway epithelial cells express TLR1 to TLR6 and TLR9, which are located either in endosomes or at the external side of the cellular plasma membrane (Muir et al., 2004; Platz et al., 2004; Mayer et al., 2007; Vareille et al., 2011). The activation of PRR triggers an intracellular pathway leading to the production and release of type I IFN from infected cells. IFNα and β (type I IFN) act similarly by binding in an autocrine or paracrine way to the heterodimer receptor IFNAR-1 and−2. Once bound to their receptor, they signal via the Janus kinase (JAK) signal transducer and activator of transcription (STAT) signaling cascade, to induce the transcription of hundreds of “IFN-stimulated genes” (ISG). The most important ISG-encoded proteins are the RNA-dependent protein kinase (PKR), 2′,5′-oligoadenylate synthetase (OAS) and RNase L, and Mx protein GTPases, which target several steps in the herpesvirus replication (Alberts et al., 2002; Sadler and Williams, 2008; Rentsch and Zimmer, 2011; Schoggins and Rice, 2011; de Weerd and Nguyen, 2012).

The early innate immune responses, guided by IFN, control the pathogen, and orchestrate the subsequent adaptive immune response against the invading pathogens. Many respiratory viruses, including alphaherpesviruses, have evolved multiple strategies to escape from the antiviral properties of IFN and persist in immuno-competent hosts by interfering with the IFN production, IFN signaling and/or the function of the IFN-induced expression of antiviral gene products (Cassady et al., 1998; Poppers et al., 2000; Yokota et al., 2001; Eidson et al., 2002; Everly et al., 2002; Lin R. et al., 2004; Lin R. J. et al., 2004; Brukman and Enquist, 2006; Melchjorsen et al., 2009; Hoffmann et al., 2015). In recent years, studies indicated that a number of EHV1 proteins modulate the early immunity to EHV1 (Ambagala et al., 2004; Van de Walle et al., 2008; Wagner et al., 2011; Wimer et al., 2011). Sarkar et al. (2015) demonstrated that the neurovirulent T953 strain shuts down IFNβ production in equine endothelial cells upon the expression of late viral proteins. Primary respiratory epithelial cells inoculated with the neurotropic Ab4 strain showed an up-regulation of mRNA expression of IFNα, which indicates the activation of the IFN response in the URT (Soboll Hussey et al., 2014). Intriguingly, recent studies of the URT demonstrated that chemokine expression varied between abortigenic and neurovirulent EHV1 strains *in vivo* and *ex vivo* (Holz et al., 2017; Zhao et al., 2017). A careful analysis of the early IFN response against EHV1 at the URT, and a comparison between abortigenic and neurovirulent variants may contribute to our understanding of the invasion kinetics of the two types of EHV1 in the URT. Indeed, an appropriate IFN response is essential to overcome viremia and severe reproductive or neurological clinical signs caused by respiratory infections (Holz et al., 2017).

Based on the phenotype-differences upon EHV1-replication and chemokine expression in the epithelium of the URT *in vivo* and *ex vivo*, we hypothesized that abortigenic EHV1 may trigger reduced amounts of IFN and/or display a decreased sensitivity to the antiviral effects of IFN on viral plaque formation at the level of the respiratory epithelium. In this work, we evaluated, by using an in-house developed *in vitro* and *ex vivo* model of the URT, the ability of abortigenic and neurovirulent EHV1 to activate the secretion of endogenous type I IFN. In addition, we analyzed whether both EHV1 phenotypes could overcome the antiviral state, induced by exogenous IFNα. Finally, we studied the effects of IFNα inhibition on replication kinetics of both EHV1 variants. Understanding the relation of both EHV1 phenotypes with IFN may have important consequences for the design of strategies to induce protective immunity and to develop new intranasal vaccination strategies.

## Materials and methods

### Virus

Different Belgian EHV1 strains were included in this study and were genotyped in the ORF30 region by the Animal Health Trust in the United Kingdom (Nugent et al., 2006). The EHV1 abortigenic strains 94P247 and 97P70 were originally isolated in 1994 and 1997 from the lungs of an aborted fetus (van der Meulen et al., 2000; Van de Walle et al., 2009). The neurovirulent 95P105 and 03P37 EHV1 strains were first isolated in 1995 and 2003 from the blood of a paralytic horse (van der Meulen et al., 2003; Garré et al., 2009). Virus stocks of all strains were used for inoculation at the 6th passage. The last passage was performed in RK-13 cells.

### Donor horses

Respiratory tissues from horses were collected post mortem in the slaughterhouse, approved by the Ethical committee of Ghent University (2018_NOPROC_01). Horses negative for ocular/nasal discharge and lung pathologies were selected. The horses were aged between 5 and 15 years old, as determined by inspection of dental incisive architecture (Muylle et al., 1996). Each experiment was conducted with tissues from three different horses. The deep part of the nasal septum and proximal part of the trachea were collected from each horse. Tissues were transported to the laboratory on ice, in phosphate-buffered saline (PBS), supplemented with 1% gentamycin, 1% penicillin-streptomycin (Gibco, Invitrogen, Paisley, UK), 1% kanamycin (Sigma-Aldrich, St. Louis, MO) and 0.5% amphotericin B (Bristol-Myers Squibb).

### Isolation and cultivation of respiratory mucosa explants

Equine respiratory mucosa explants were obtained as described previously (Vandekerckhove et al., 2010). Briefly, mucosa was stripped from the cartilage of the deep part of the nasal septum and the proximal trachea. Mucosa was divided into equal explants of 0.5 cm^2^ and placed upwards on fine-meshed gauzes. Explants were cultured in serum-free medium at the air-liquid interface at 37°C and 5% CO_2_.

### Cells

#### Isolation and cultivation of equine respiratory epithelial cells

The isolation and culture of equine respiratory epithelial cells (EREC) were adapted from the protocol described by Quintana et al. (2011). Briefly, equine tracheal tissues were washed twice with cold Dulbecco's phosphate-buffered saline (DPBS) to remove red blood cells. Epithelial cells were isolated by an enzymatic digestion, using gentle agitation in calcium and magnesium-free minimal essential medium (MEM) containing 1.4% pronase (Roche Applied Science, Indianapolis, IN) and 0.1% deoxyribonuclease I (Sigma-Aldrich, St. Louis, MO). Tissues were incubated during 48 h with the enzyme mix. Cells were cultured in a plastic uncoated petri dish for 6 h in DMEM/F12 containing calcium and magnesium-free MEM, 1% penicillin-streptomycin (Gibco, Invitrogen, Paisley, UK) and 2.4 μg/ml insulin (Sigma-Aldrich, St. Louis, MO) to reduce fibroblast contamination. EREC were stored in liquid nitrogen at a density of 2 million cells per cryovial until further use. For culture, we seeded the EREC into type IV collagen (Sigma-Aldrich, St. Louis, MO) coated transwell cell culture wells (Costar, Corning, Fisher Scientific, Fair Lawn, NJ) in DMEM/F12, containing 5% non-heat-inactivated fetal bovine serum (Gibco, Invitrogen, Paisley, UK), 1% calcium and magnesium-free MEM, 1% penicillin-streptomycin (Gibco, Invitrogen, Paisley, UK) and 0.5% amphotericin B (Biowhittaker, Walkersville, MD). After 24 h of culture, we removed the medium and cultivated the cells at an air-liquid interface in epithelial cell medium containing DMEM/F12 supplemented with 2% Ultroser G (Pall Life Sciences, Pall Corp., Cergy, France), 1% penicillin-streptomycin (Gibco, Invitrogen, Paisley, UK) and 0.5% amphotericin B (Biowhittaker, Walkersville, MD). EREC were incubated in a humidified incubator at 37°C, 5% CO_2_ until differentiated.

#### Rabbit kidney epithelial (RK-13) cells

RK-13 cells were purchased from the American Type Culture Collection (ATCC, Manassas, Virginia, USA) and were used in this study to quantify EHV1 replication. RK-13 cells were maintained in Modified Eagle's medium (MEM, Gibco, Invitrogen, Paisley, UK) supplemented with antibiotics and 5% FCS (Gibco, Invitrogen, Paisley, UK). Extracellular virus titers were determined at different time points post inoculation.

#### Analysis of viability

An *in situ* Cell Death Detection Kit (Fluorescein) based on Terminal deoxynucleotidyl transferase mediated dUTP Nick End Labeling (TUNEL) was obtained from Roche (Mannheim, Germany) and used to detect DNA fragmentation induced by apoptotic signaling cascades. The assay was performed on mucosa explants pre-treated with recombinant equine IFNα or an IFN signaling pathway inhibitor. Cells were analyzed for incorporation of dUTP with a fluorescence microscope (Leica DM RBE microscope, Leica Microsystems GmbH, Heidelberg, Germany). The number of TUNEL-positive cells was evaluated in five randomly chosen fields of 100 cells in epithelium as well in the lamina propria.

### Pre-treatment with recombinant equine IFNα or an IFN inhibitor prior to and during EHV1 infection

At 12 h of culture, explants were taken from their gauzes and placed in a 24-well plate with the epithelial surface upwards. Warm serum-free medium was used to wash them twice. Explants were inoculated based on the agarose-model, described by Vairo et al. (2013). Briefly, a new 24-well plate was embedded with 1 ml of a solution containing 50% of sterile 3% agarose (low gelling temperature; Sigma-Aldrich, St. Louis, MO) and 50% of 2X medium (50% 2X DMEM and 50% 2X F12 supplemented with 2 μg/mL gentamicin, 0.2 mg/mL streptomycin and 200 U/mL penicillin). Explants were placed on top of the solified agarose layer with the epithelium upwards. The lateral surfaces of the mucosa were covered by additional agarose. Subsequently, explants were inoculated with 1 ml inoculum containing 10^6.5^ TCID_50_ of 97P70, 94P247, 03P37, or 95P105 EHV1 strain. After incubation, explants were washed twice in warm serum free medium and transferred to their gauzes. At 10, 24, 48, and 72 hpi mucosa explants were collected and embedded in methylcellulose medium (Methocel® MC, Sigma-Aldrich, St. Louis, MO) and frozen at −70°C. Supernatant was collected and stored at −70°C until further use. As positive control, explants were pre-treated during 18 h with recombinant equine IFN alpha (rEqIFNα) [(Kingfisher Biotech, Inc. (Saint Paul, MN)] and collected at 48 hpi.

EREC were inoculated *in vitro* with EHV1 strains 97P70, 94P247, 03P37, and 95P105 at a MOI of 1 in 200 μl epithelial cell culture medium for 1 h at 37°C with 5% CO_2_. Next, cells were gently washed twice in DMEM/F12, to remove the inoculum and further incubated in fresh medium. Mock inoculations were carried out in parallel. At 10, 24, and 48 hpi, supernatants were collected for quantification of viral replication and IFN secretion. EREC were then fixed in 100% methanol for 20 min at −20°C.

Where mentioned, 0, 10, 100, 1000 laboratory units per ml (U/ml) rEqIFNα [Kingfisher Biotech, Inc. (Saint Paul, MN)], were added 18 h before inoculation and maintained throughout the inoculation and cultivation of the explants and EREC. The inhibitor of the IFN response Ruxolitinib (Rux) (Selleck Chemicals) was prepared as 10 mM stocks in dimethyl sulfoxide (DMSO) and used at a concentration of 0.04, 0.4, and 4 μM. Where indicated, mucosa explants and EREC were incubated with Rux or an equivalent volume of DMSO during 2 h (37°C, 5% CO_2_) prior to infection and maintained throughout inoculation and cultivation of the mucosa explants and EREC.

The concentration of rEqIFNα, Rux, or DMSO used in this study did not decrease cell viability, as determined by TUNEL staining. Mucosa explants pre-treated with rEqIFNα, Rux, or DMSO were collected at 48 hpi.

### Indirect immunofluorescence staining

#### EHV1 proteins

At different time points, consecutive cryosections of 16 μm were made of the frozen explants. The frozen sections were mounted on 3-aminopropyltriethoxysilane (Sigma-Aldrich, St. Louis, MO) coated slides. They were fixed in 100% methanol for 20 min at −20°C, and then washed with DPBS. Late viral proteins were stained with biotinylated equine polyclonal anti-EHV1 IgG antibody (1:20 in DPBS) (van der Meulen et al., 2003), followed by streptavidin FITC (1:200 in DPBS) (Molecular Probes, Eugene, OR). Subsequently, the BM of the explants was stained by incubation with a mouse monoclonal anti-collagen VII IgG1 antibody (clone LH7.2; 1:50 in DPBS) (Sigma-Aldrich, St. Louis, MO), followed by a Texas Red®-conjugated goat anti-mouse IgG antibody (1:200 in DPBS) (Molecular Probes, Eugene,OR). Antibodies were incubated for 1 h at 37°C. Determinations of viral plaque numbers and plaque latitude were based data obtained from 50 consecutive cryosections.

#### Interferon alpha

At 48 hpi, mucosa explants and EREC were pre-treated with 10 μg/ml Brefeldin A (Sigma-Aldrich, St. Louis, MO) for 2 h and 30 min, respectively. Fifty consecutive cryosections of 16 μm were made of the frozen explants and fixed in 100% methanol for 20 min at −20°C. Viral proteins were stained by incubation with biotinylated equine polyclonal anti-EHV1 IgG antibody (1:20 in DPBS) (van der Meulen et al., 2003), followed by secondary streptavidin-TR® (1:200 in DPBS) (Molecular Probes, Eugene, OR). IFNα was detected by incubation of the cultures with a rabbit polyclonal anti-equine IFNα1 IgG antibody (1:50 in DPBS) (Kingfisher Biotech, Inc., Saint Paul, MN), followed by secondary FITC®-labeled goat anti-rabbit IgG antibody (1:100 in DPBS) (Molecular Probes, Eugene, OR). EREC were stained similarly as mucosa explants.

### Confocal microscopy

Immunofluorescence of cryosections and EREC were analyzed by confocal microscopy (Leica TCS SP2 Laser Scanning Spectral Confocal System; Leica Microsystems). A Gre-Ne 543 nm laser was used to excite Texas Red-fluorochromes. An Argon 488 nm laser excited FITC-fluorochromes.

### Bioassay for determining IFN antiviral activity

Supernatants of EHV1- and mock-inoculated explants were harvested at 10, 24, 48, and 72 hpi. Type I IFN bioactivity was determined by a cytopathic effect (CPE) reduction assay based on vesicular stomatitis virus (VSV) and Madin-Darby bovine kidney (MDBK) cells (La Bonnardiere and Laude, 1981). MDBK cells were seeded in 96-well micro titer plates in Dulbecco's modified Eagle Medium (DMEM) with 5% FCS, 1% sodium pyruvate, 1% penicillin-streptomycin solution and 1% gentamycin. After overnight incubation at 37°C, 5% CO_2_, medium was removed and serial twofold dilutions of samples were added to the confluent cells. Following 18 h incubation (37°C, 5% CO_2_), 50 μl of VSV was added to the samples and virus control wells, at a concentration resulting in complete CPE after 48 h. To the cell control wells, only 50 μl of medium was added. Following 48 h incubation, medium was aspirated and 50 μl of 0.1% neutral red solution was added to the cells during 1 h at 37°C in 5% CO_2_. Next, cells were rinsed, air-dried and 150 μl of dissolving solution (50 μl sodium lauryl sulfate (SDS), 100 μl 0.2 M HCl in H_2_O) was added. The absorbance of neutral red solution at 492 nm was determined with a micro-plate reader. The IFN titer was calculated as the reciprocal of the last IFN dilution causing 50% inhibition of virus-induced CPE and was expressed as IFN units per volume. Recombinant EqIFNα [Kingfisher Biotech, Inc., (Saint Paul, MN)] with a titer of 18 x 10^3^ U/ml was run in each assay.

### Quantification of IFNα levels with ELISA

IFNα quantification was assessed by an ELISA assay using the commercial Quantikine immunoassay kit (GSI Equine-IFN alpha, Genorise Scientific, Inc., USA) according to the manufacturer's instructions. Briefly, the samples were added for 1 h at room temperature to 96-well microtiter plates, coated with primary antibody. After washing, the secondary antibody conjugate with peroxidase was added to each well and incubated. The concentration of the cytokines was determined by the intensity of the color measured spectrophotometrically using a micro-plate reader.

### Virus titration

The extracellular viral titer was determined in supernatant from mucosa explants and EREC. Virus titers were assessed by a 50% tissue culture infective dose assay using RK-13 cells. The 50% end-point was calculated according to the method of Reed and Muench (Reed and Muench, 1938).

### Statistical analyses

Analyzed data for statistical significance were subjected to a multiple-way analysis of variance (ANOVA). The Scheffé-test was used as a *post-hoc* test. If the assumption of equal variables was not fulfilled with the Levene's test, the data were log-transformed prior to ANOVA. Normality of the residuals was verified by the use of the Shapiro-Wilk test. A Kruskall-Wallis' test, followed by a Mann-Whitney's *post-hoc* test was performed when variables remained unequal or when normality was not achieved after log-transformation. Differences in results with *p* < 0.05 were considered significant. The data shown represent means ± SD of independent experiments. Data were statistically evaluated with IBM SPSS Statistics for Windows, version 23.0 (IBM Corp, Armonck, NY, USA).

## Results

### EHV1 activates IFN production in equine respiratory mucosa explants and EREC

First, EHV1 replication was evaluated in the mucosa explants and EREC from the URT by analyzing the number of viral plaques and plaque latitudes after IF staining and by fluorescence microscopy (see Supplementary Figures 1A,B). Nasal and tracheal mucosa explants and EREC were (mock) inoculated with 1 ml of EHV1 97P70, 94P247, 03P37, or 95P105 strains containing 10^6.5^ TCID_50_ virus. At 10, 24, 48, and 72 hpi, the supernatants of mucosa explants were collected and explants were fixed. EREC and EREC-supernatants were collected at 10, 24, and 48 hpi. In the explants from the nasal septum, the number, and latitude of the viral plaques increased in time for both EHV1 phenotypes. At 72 hpi, we observed a trend of more and larger viral plaques induced by the abortigenic variants, compared to the neurovirulent strains, albeit the differences were not statistically significant. This corresponds with the observations described by Vandekerckhove et al. (2010) and Gryspeerdt et al. (2010). In EHV1-inoculated tracheal mucosa explants, the abortigenic strains showed more plaques compared to the neurovirulent strains at 72 hpi. However, the difference in plaque latitude between the two phenotypes was less pronounced. In EHV1-inoculated EREC, no differences between the two types of strains could be observed. These results imply a tissue specific replication potential of EHV1. Next, viral replication was evaluated by virus titration of the supernatant (see Supplementary Figure 1C). Both abortigenic and neurovirulent EHV1 variants produced similar levels of new virus particles and no significant differences were observed between both types of variants. However, we detected slightly, but not significant higher virus titers in supernatant derived from tracheal mucosa explants, compared to nasal mucosa explants (*p* = 0.062). Taken together, mucosa explants and EREC are suitable models to investigate the role of IFN in respiratory EHV1 infections.

**Figure 1:**
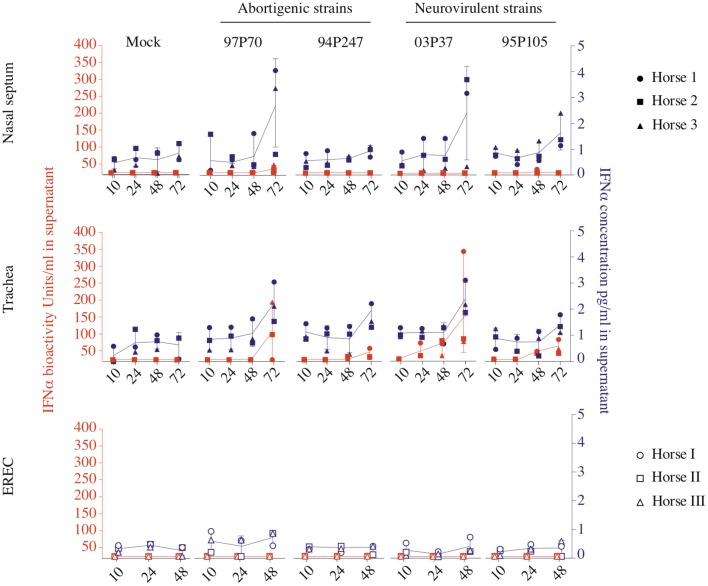
Type I IFN concentration (blue) and bioactivity (orange) in equine respiratory mucosa explants and equine respiratory epithelial cell (EREC) cultures. Each symbol indicates one horse. Symbols in blue and orange represent IFNα concentration and bioactivity detected by ELISA and CPE reduction assay, respectively. Nasal (upper panel), tracheal (middle panel) mucosa explants, and EREC (lower panel) were inoculated with two abortigenic (97P70 and 94P247) and two neurovirulent (03P37 and 95P105) EHV1 strains. At 10, 24, 48, and 72 hpi, supernatant was collected to assess IFNα concentration and bioactivity. Experiments were performed with mucosa explants of 3 horses (horse 1●, 2 ■, and 3 ▲) and EREC of 3 other horses (horse I ○, II □, and III △).

Therefore, the type I IFN production was concurrently analyzed in the URT after EHV1 infection. Equine IFNα concentration and bioactivity were assessed in the supernatants by ELISA and by a CPE reduction assay based on VSV and MDBK cells. Mucosa explants pre-treated during 18 h with rEqIFNα (100 U/ml) were used as positive control and were collected at 48 hpi. Determining the IFNα concentration in the supernatant of mucosa explants by ELISA, showed that all EHV1 strains triggered IFNα production starting from 48 hpi, compared to non-inoculated explants (Figure 1, blue symbols). The IFNα concentration significantly increased from 48 to 72 hpi for all EHV1 strains (*p* < 0.05) in nasal and tracheal mucosa explants (Figure 1, upper and middle panel). In addition, in EREC, IFNα was constitutively present at all time points, however no significant increase in time was observed (Figure 1, lower panel).

To analyze whether the produced IFNα is bioactive, a CPE reduction assay was performed, as shown in orange in Figure 1. Bioactive IFNα could be detected in the supernatant of EHV1 inoculated explants, while the IFNα bioactivity did not reach the limit of detection of 20 U/ml in mock-inoculated explants. Positive controls showed high IFNα bioactivity in nasal (556 ± 166 U/ml) and tracheal (399 ± 380 U/ml) mucosa explants (data not shown). Surprisingly, only nasal mucosa explants inoculated with the 97P70 abortigenic strain (33 ± 14 U/ml) and 95P105 neurovirulent strain (20 ± 2 U/ml), showed a low-level increase of IFNα bioactivity starting from 48 hpi (Figure 1, upper panel). In tracheal mucosa explants, similar levels (45 ± 44 U/ml) of IFNα were detected for all strains at 48 hpi. From 48 to 72 hpi the IFNα expression increased to 100 ± 109 U/ml for abortigenic 97P70 and 94P247 and 99 ± 120 U/ml for the neurovirulent 03P37 and 95P105 strains. These results of tracheal mucosa explants correspond with the IFNα concentration determined by ELISA. In contrast, the IFNα concentrations in nasal mucosa explants detected by ELISA did not correspond with the measured IFN bioactivity. This suggests that not all IFNα secreted by nasal mucosa, is bioactive. Furthermore, bioactive IFNα secreted by EHV1-inoculated tracheal mucosa explants was significantly higher, compared to EHV1-inoculated nasal mucosa explants (*p* < 0.05). These results imply that the production of IFNα in the nasal cavity is strictly regulated in order to orchestrate the beneficial and negative effects of IFNα in the host. Based on the results obtained by ELISA and by CPE-reduction assay we conclude that both abortigenic and neurovirulent EHV1 strains trigger IFNα production to the same level.

**Figure 2:**
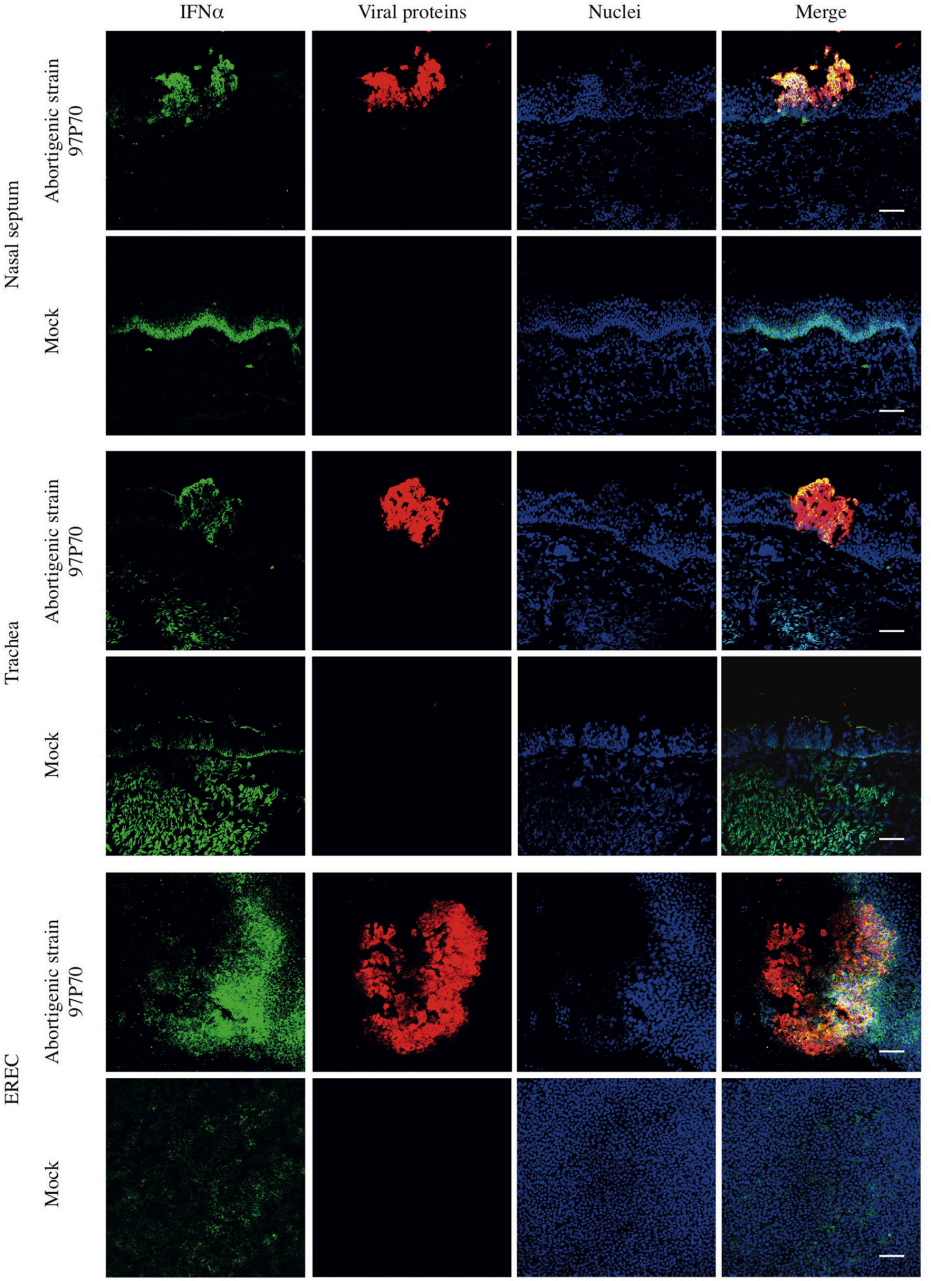
Double immunofluorescence staining of EHV1 viral proteins and IFNα in equine respiratory mucosa explants and equine respiratory epithelial cell (EREC) cultures. At 48 hpi, mucosa explants and EREC were pretreated with Brefeldin A for 2 h and 30 min, respectively. EHV1-induced plaques (red) and IFNα (green) in nasal and, tracheal mucosa explants and EREC were visualized by immunofluorescence staining. IFNα expression is highly co-localized with viral plaques. Non-inoculated explants and EREC show a base-line expression of IFNα. Nuclei were counterstained with Hoechst (blue). Bars, 75 μm.

Finally, the expression of IFNα in mucosa explants was confirmed by immunofluorescence (IF) staining. As shown in Figure 2, constitutive expression of IFNα was observed in the basal part of respiratory epithelium in mock-inoculated nasal and tracheal mucosa explants at 48 hpi. In tracheal mucosa explants, secreted IFNα could also be observed at the apical side of the epithelium. Upon inoculation with abortigenic or neurovirulent EHV1 strains, the expression of IFNα co-localized with the EHV1 infected epithelial cells and was expressed in the apical and basal part of the respiratory epithelium, in contrast to control samples. No significant differences in IFNα expression or co-localization with infected epithelial cells were observed between the two types of EHV1 strains (data not shown). IF staining of EREC demonstrated a low-level constitutive expression of IFNα in non-inoculated EREC at 48 hpi. The expression of IFNα increased and co-localized with EHV1 viral proteins in EREC over time pi.

Taken together, these data demonstrate that the expression of type I IFN is up regulated equally by both abortigenic and neurovirulent EHV1 phenotypes in respiratory epithelium. IFNα concentration in nasal and tracheal mucosa was similar, while the IFNα bioactivity appears to be hampered in the nasal cavity.

### EHV1 is susceptible to rEqIFNα in respiratory mucosa explants and EREC

In the next experiments, we investigated whether both EHV1 variants could overcome the IFN-induced antiviral state, by treatment of the respiratory mucosa explants and EREC with exogenous rEqIFNα (0, 10, 100, and 1000 U/ml) 18 h prior to and during abortigenic and neurovirulent EHV1 infection. Cell viability determined by TUNEL staining did not show any significant differences between rEqIFNα treated and non-treated mucosa explants or EREC (>95% TUNEL negative cells), suggesting that any inhibition of EHV1 was not the result of IFN-mediated cytotoxicity (see Supplementary Table 1).

**Figure 3:**
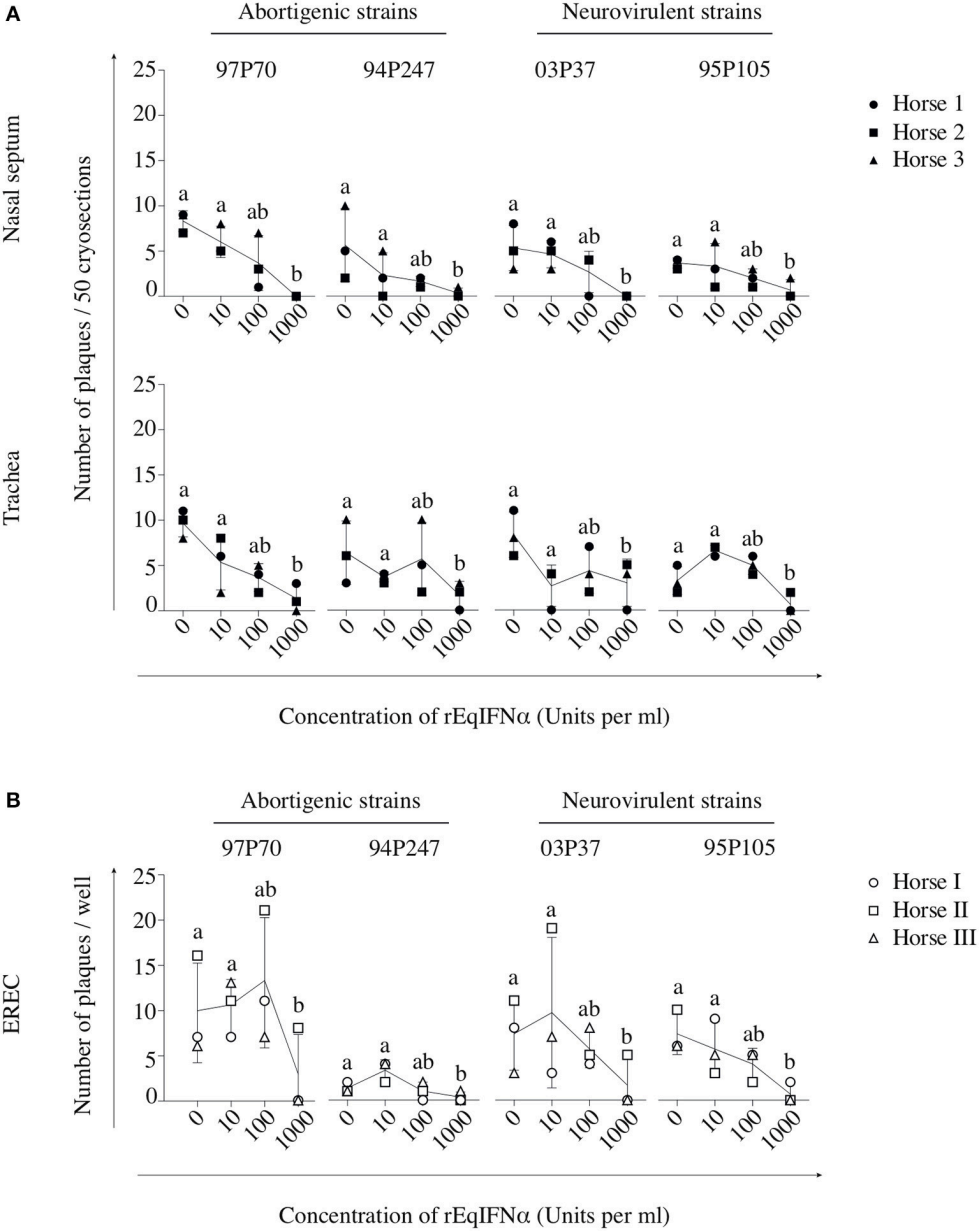
Pretreatment with rEqIFNα reduces the number of EHV1 viral plaques in equine respiratory mucosa explants and equine respiratory epithelial cell (EREC) cultures. Each symbol represents one horse. **(A)** Mucosal explants (nasal and tracheal) and **(B)** EREC were treated with 0, 10, 100, and 1000 U/ml rEqIFNα 18 h prior to and during EHV1-inoculation with two abortigenic (97P70 and 94P247) and two neurovirulent (03P37 and 95P105) variants. Explants and supernatants were collected at 48 hpi. Different letters represent significant differences (*p* < 0.05) in the number of viral plaques. Experiments were performed on mucosa explants of 3 horses (horse 1 ●, 2 ■, and 3 ▲) and EREC of 3 other horses (horse I ○, II □, and III △).

**Figure 4:**
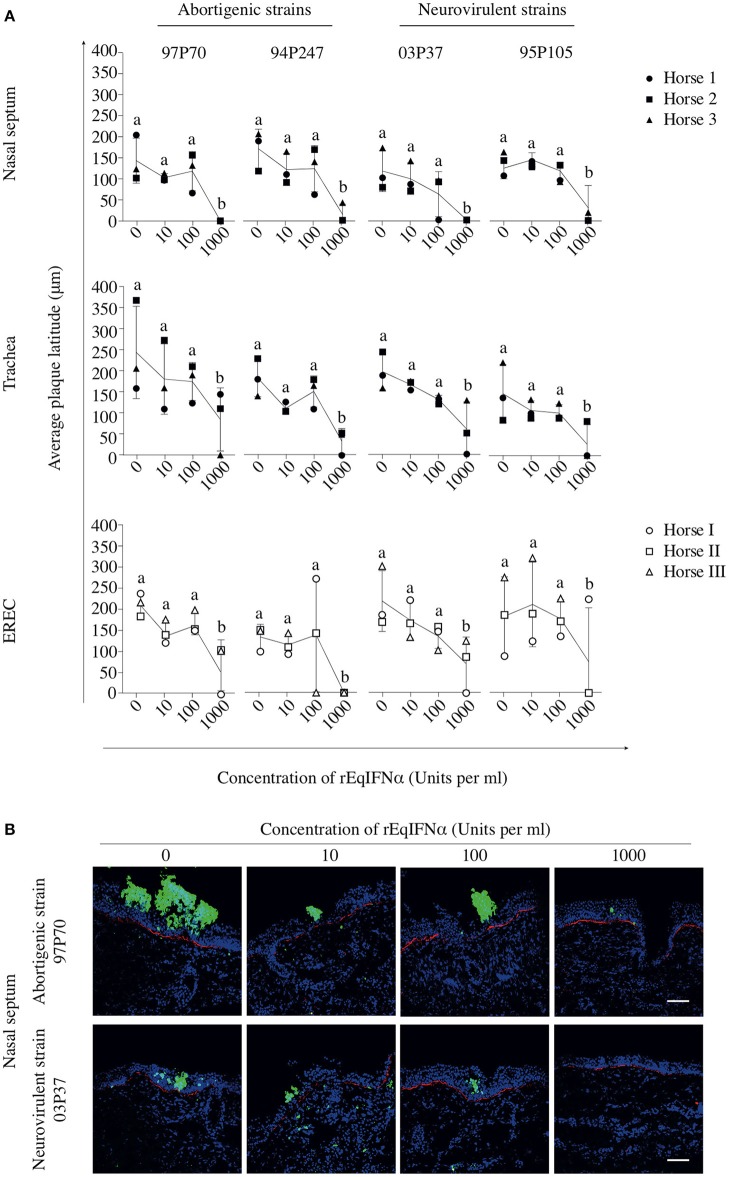
Pretreatment with rEqIFNα reduces EHV1 plaque latitudes in equine respiratory mucosa explants and equine respiratory epithelial cell (EREC) cultures. **(A)** Graphs represent plaque latitudes in rEqIFNα-pretreated nasal (upper panel), tracheal (middle panel) mucosa explants and EREC (lower panel) at 48 hpi. Different letters represent significant differences (*p* < 0.05) in viral plaque latitudes. **(B)** Immunofluorescence in plaques induced by abortigenic and neurovirulent strains (green) and staining of the basement membrane (red) in nasal mucosa explants are shown. Nuclei were counterstained with Hoechst (blue). Bars, 75 μm. Experiments were performed in triplicate.

**Figure 5:**
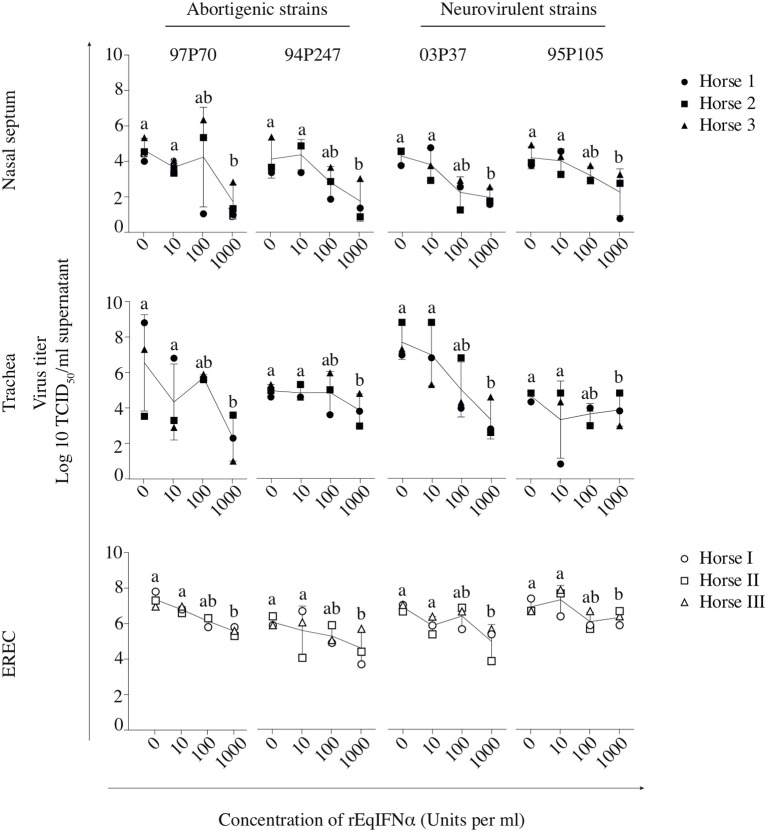
Replication of EHV1 in equine respiratory mucosa explants and equine respiratory epithelial cell (EREC) cultures following treatment with rEqIFNα. The efficacy of rEqIFNα treatment was measured by the extracellular virus titer of IFN-treated explants and cells. Each symbol represents one horse. Experiments were performed on 3 individual horses. Different horses were used to isolate mucosa explants (horse 1●, 2 ■, and 3 ▲) and EREC (horse I ○, II □, and III △). Different small letters represent significant differences (*p* < 0.05) in virus titers.

In abortigenic EHV1-inoculated nasal mucosa explants, the number of plaques decreased from 8 ± 1 of 97P70 plaques and 6 ± 4 of 94P247 plaques at 0 U/ml to 4 ± 3 and 2 ± 1 at 100 U/ml, respectively. At 1000 U/ml no 97P70 plaques were observed and the number of 94P247 plaques was significantly reduced to 0 ± 1 (*p* < 0.05) (Figure 3A, upper panel). A similar reduction was observed in tracheal mucosa explants (Figure 3A, lower panel) and EREC (Figure 3B). Nasal mucosa explants inoculated with the neurovirulent EHV1 strain 03P37 showed a decrease from 10 ± 2 to 1 ± 2 of plaques when rEqIFNα concentration increased from 0 to 1000 U/ml, respectively. A similar reduction was seen for the 95P105 strain from 4 ± 1 to 1 ± 1 of plaques with increasing rEqIFNα concentration (Figure 3A, upper panel). Analogous results were observed in tracheal mucosa explants (Figure 3A, lower panel) and EREC (Figure 3B). As shown in Figure 4A, a decrease in plaque latitude was observed in both respiratory mucosa explants and EREC with increasing rEqIFNα concentration. In nasal mucosa explants, plaque latitudes of 152 ± 86 μm for 97P70 and 170 ± 47 μm for 94P247 were measured at 0 U/ml rEqIFNα. This latitude decreased to 103 ± 33 μm for 97P70 and 91 ± 83 μm for 94P247 strains at 10 U/ml. However, at 100 U/ml an increase in plaque latitude was observed in two out of three horses for both 97P70 (132 ± 52 μm) and 94P247 (89 ± 43 μm) abortigenic strains, compared to 10 U/ml. At 1000 U/ml, plaque latitudes of 97P70 and 94P247 significantly dropped to 0 ± 0 μm and to 14 ± 24 μm, respectively (*p* < 0.05) (Figure 4A, upper panel). Similar results were observed for tracheal mucosa explants and EREC inoculated with the abortigenic strains. Here, again, an increase in abortigenic EHV1 plaque latitude at 100 U/ml compared to 10 U/ml was seen for two out of three horses (Figure 4A, middle and lower panel). Similarly, plaque latitudes decreased for both neurovirulent strains from 119 ± 51 μm to 0 ± 0 μm for the 03P37 strain and 147 ± 69 μm to 27 ± 47 μm for 95P105 strain with increasing rEqIFNα concentration. In contrast to the abortigenic strains, the neurovirulent strains did not show a similar trend in plaque latitudes at a concentration of 100 U/ml when compared to 10 U/ml. Figure 4B shows representative confocal images of nasal mucosa explants, treated with 0, 10, 100, and 1000 U/ml of rEqIFNα prior to abortigenic and neurovirulent EHV1 infection. Next, we quantified EHV1 replication in the presence or absence of rEqIFNα, by determining extracellular virus titers by titration of the supernatants of inoculated respiratory mucosa explants and EREC at 48 hpi on RK-13 cells. We first demonstrated that rEqIFNα that may be present in the supernatants did not have a direct effect on EHV1 replication in RK-13 cells that were used for virus titrations (see Supplementary Figure 2). IFN-pretreated and EHV1-inoculated respiratory mucosa explants showed a concentration-dependent decrease in extracellular virus titers for both abortigenic and neurovirulent EHV1 variants. In addition, at 100 U/ml, for two out of three horses, the abortigenic 97P70 EHV1 strain showed a 2-log increase of virus titer, compared to 10 U/ml. And one out of three horses showed a 1-log virus titer increase for the 94P247 EHV1 strain at this concentration of IFN in tracheal mucosa explants. In general, this was not observed in either of the two EHV1 neurovirulent strains (Figure 5). Although this increase was not statistical significant due to the large biological variation between the individual horses, it does seem to correspond with the increased plaque latitudes observed at 100 U/ml. At a high concentration of 1000 U/ml rEqIFNα, a significant lower virus titer was observed in nasal and tracheal mucosa explants compared to lower concentrations (*p* < 0.05) for all EHV1 strains (Figure 5, upper and middle panel). Similar results were observed in EREC when pretreated with 0, 10, 100, and 1000 U/ml rEqIFNα. However, no increase in virus titer was observed at 100 U/ml (Figure 5, lower panel).

Taken together, we conclude that both EHV1 phenotypes are susceptible to the IFN-induced antiviral state in respiratory mucosa explants and EREC, in a largely dose-dependent manner. However, the abortigenic EHV1 strains show a window of IFN concentration (10–100 U/ml) where increasing IFN concentration does not always correlate with decreased viral replication and spread.

### Inhibition of the IFN response enhances EHV1 replication *ex vivo*

**Figure 6:**
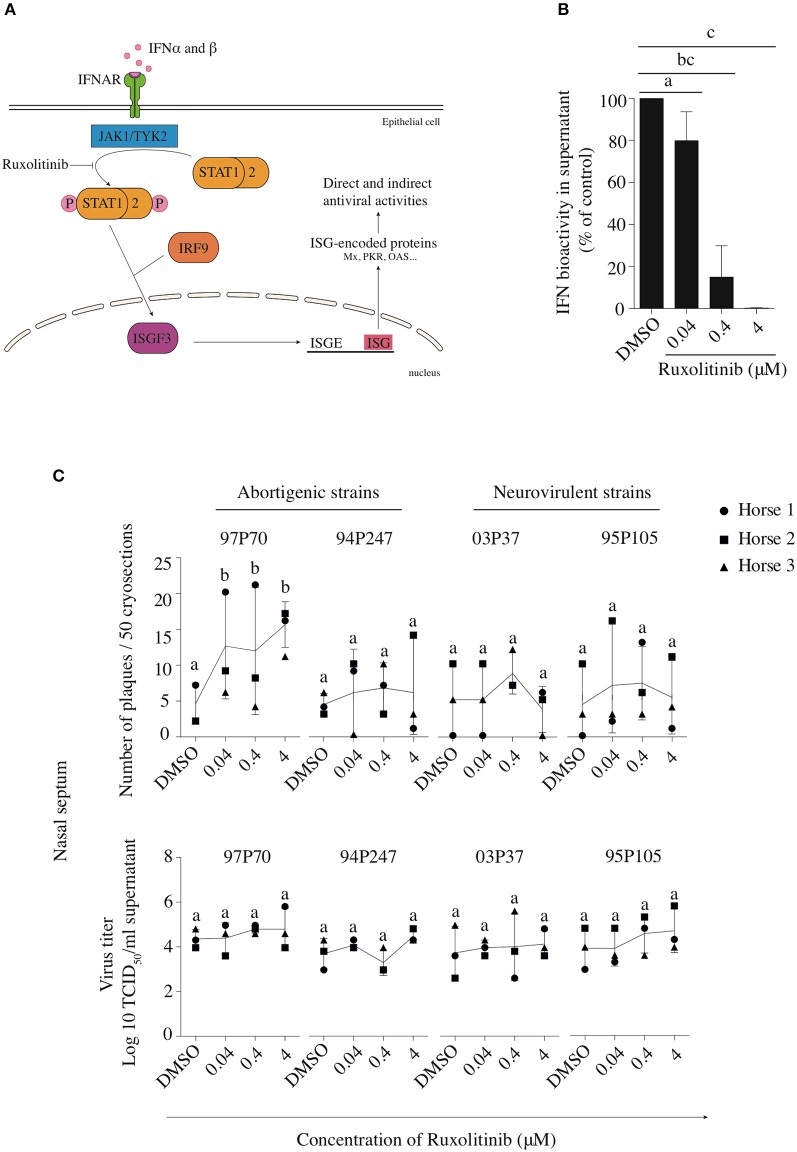
Effects of Rux on the replication of abortigenic and neurovirulent EHV1 strains in nasal mucosa explants. **(A)** Rux inhibits the JAK/STAT signaling pathway by hindering the phosphorylation of STAT-1 and-2. **(B,C)** Mucosa explants from the nasal septum are used to demonstrate the effects of the IFN-signaling inhibitor Rux. Explants were pretreated with 0.04, 0.4, or 4 μM of Rux or equivalent DMSO concentrations for 2 h prior to EHV1 inoculation. At 48 hpi, explants were collected and fixed for immunofluorescence straining to detect viral proteins. **(B)** The efficacy of Rux-treatment was calculated from the IFN bioactivity in treated versus non-treated explants. **(C)** Number of viral plaques in the presence or absence of Rux were counted and demonstrated in the upper panel. The extracellular virus titer was determined in the supernatant and shown in the lower panel. Each symbol represents one horse (horse 1●, 2 ■, and 3 ▲). Different small letters represent significant differences (*p* < 0.05) in virus titers.

To confirm that the replication of the neurovirulent EHV1 variants in the URT is affected more substantially by IFNα compared to the abortigenic EHV1 variants, we performed assays using the inhibitor Rux that targets JAK1, a major component of the IFN signaling pathway. Figure 6A shows the area of action of Rux in the JAK/STAT pathway. Since the primary site of EHV1-replication is the nasal mucosa, all further experiments were conducted in mucosa explants isolated from the deep part of the nasal septum.

Based on Stewart et al. (2014), we pretreated mucosa explants with increasing concentrations of Rux (0.04, 0.4 and 4 μM) or DMSO (control) for 2 h prior to viral inoculation. Explants remained in the presence of Rux or DMSO throughout the cultivation. Explants were collected and fixed at 48 hpi. Rux (or DMSO alone) did not affect cell viability in nasal mucosa explants (see Supplementary Table 2). Also, to determine whether Rux potentially could directly enhance EHV1 replication, independently from the IFN signaling pathway, a virus titration assay was performed on RK-13 cells in the presence or absence of the inhibitor. We found that Rux did not directly enhance EHV1 replication in RK-13 cells (see Supplementary Figure 3). Rux directly inhibits the IFN signaling pathway as confirmed by testing the IFN bioactivity of supernatant of mucosa explants on VSV inoculated MDBK cells pretreated or not with this inhibitor (Figure 6B).

Pretreatment of nasal mucosa explants with different concentrations of Rux did not affect the number of plaques of 94P247, 03P37, and 95P105 EHV1 strains, compared to non-treated cells, while the 97P70-abortigenic strain showed a significant increase in number of plaques (*p* < 0.05)(Figure 6C, upper panel).

To quantify EHV1 replication in the Rux-treated explants, extracellular virus titers were determined at 48 hpi and were compared to virus titers in control-treated explants. No significant increase was observed in virus titers in Rux-treated explants (Figure 6C, lower panel).

**Figure 7:**
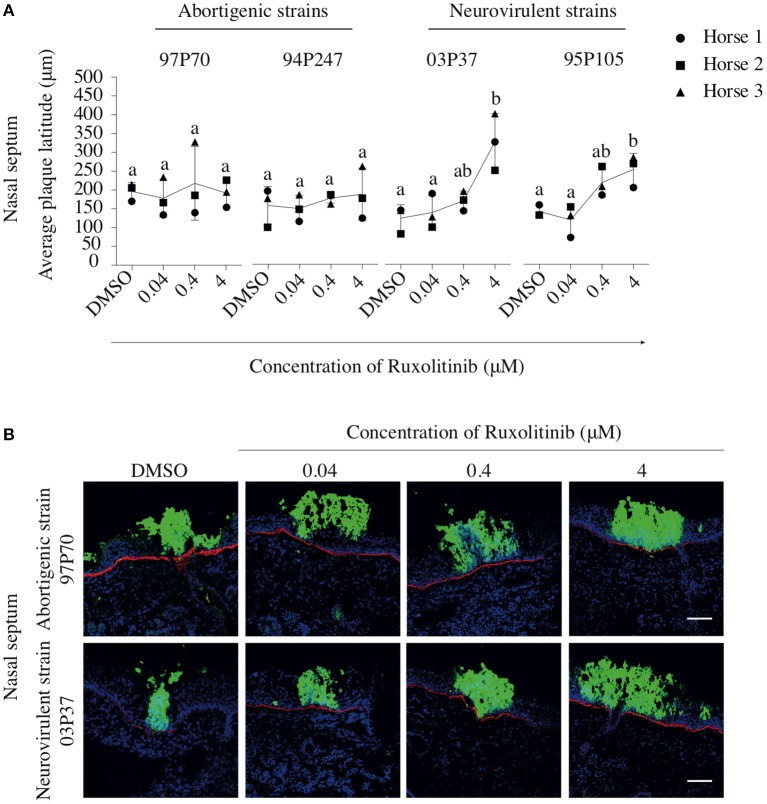
Effects of Rux on the lateral spread of abortigenic and neurovirulent EHV1 strains in nasal mucosa explants. **(A)** Mucosa explants from the nasal septum were treated with Rux for 2 h at 37°C, followed by EHV1 inoculation in the presence of Rux or DMSO (control). At 48 hpi, explants were collected and the latitudes of the viral plaques were measured. Each symbol represents one horse. Different small letters represent significant differences (*p* < 0.05) in virus titers. **(B)** Immunofluorescent pictures of Rux-treated or non-treated mucosal explants inoculated with the 97P70 abortigenic and 03P37 neurovirulent EHV1 strains. Plaque latitudes of the presented strains are representative for the plaque latitude of the abortigenic and neurovirulent phenotype. Bars, 75 μm.

In addition, the effect of Rux treatment on viral plaque latitudes was analyzed and is shown in Figure 7A. Both abortigenic EHV1 strains did not show a Rux-mediated increase in plaque latitude, compared to non-treated explants. Indeed, plaque latitudes of 97P70 and 94P247 did not show statistically significant differences at the concentration of 0.04 μM (167 ± 51 and 154 ± 35 μm) compared to non-treated explants (184 ± 23 and 162 ± 51 μm). Also, no concentration-dependent raise of plaque latitudes was present when Rux concentration increased to 0.4 μM (206 ± 97 and 181 ± 13 μm) and 4 μM (180 ± 36 and 191 ± 69 μm). However, Rux-treated explants inoculated with the neurovirulent EHV1 strains showed a dose-dependent increase in plaque latitudes, compared to non-treated explants. This indicates an enhanced lateral spread of the neurovirulent strains when the IFN signaling pathway is suppressed. Indeed, although the 03P37 and 95P105-inoculated mucosa explants showed no significant differences between 0.04 μM Rux-treated tissues (138 ± 46 and 118 ± 42 μm) and non-treated tissues (123 ± 36 and 143 ± 84 μm), plaque latitudes of 03P37 and 95P105 increased when Rux concentration increased from 0.04 to 0.4 μM (169 ± 26 and 214 ± 42 μm). Another 1-log raise of Rux concentration (4 μM) resulted in a three-fold increase (325 ± 75 μm) and two-fold increase (221 ± 72 μm) of plaque latitude for the 03P37 and 95P105 strain respectively, compared to control (*p* < 0.05). Corresponding representative confocal images are shown in Figure 7B.

In summary, these results show that viral plaques expand when the IFN signaling pathway is inhibited in mucosal explants inoculated with the EHV1 neurovirulent strains but not with the EHV1 abortigenic variants. The number of neurovirulent viral plaques caused by the neurovirulent strains remained unaltered in the presence of the inhibitor, while the number of plaques increased in one of the two EHV1 abortigenic strains.

## Discussion

The pathogenesis of respiratory alphaherpesviruses begins with replication of the virus in the epithelium of the URT, which can result in respiratory disorders. During the early stage of infection, the outcome of herpesvirus infections is dependent on the balance between virus spread and the initiation of innate immune responses, such as the IFN response (Melchjorsen et al., 2009). Several studies already demonstrated that alphaherpesviruses have developed specific mechanisms to overcome the IFN response (Härle et al., 2002; Melchjorsen et al., 2009; Johnson and Knipe, 2010). Herpes simplex virus 1 (HSV1), an important alphaherpesvirus in humans, has evolved many mechanisms to interfere with the IFN antiviral response. A few examples are the interference of ICP34.5 with the phosphorylation of eIF2α, and of US11 with OAS. EHV1 is a close relative of human varicella-zoster virus and HSV and is associated with symptoms varying from respiratory problems to neonatal foal disease, abortion and paralysis in *Equidae* (Frampton et al., 2007). Remarkably, a recent increase in the incidence of equid herpes myeloencephalopathy (EHM) outbreaks has suggested a change in the virulence of the virus (Pusterla et al., 2008; Lunn et al., 2009; Wagner et al., 2011). The prevalence of EHV1-induced reproductive or neurological outbreaks is associated with a single nucleotide polymorphism in the catalytic subunit of the viral DNA polymerase (Nugent et al., 2006; Goodman et al., 2007). Interestingly, plaque sizes of abortigenic strains are significantly larger at 48 and 72 hpi compared to plaque sizes of neurovirulent strains, the same time points at which type I IFN secretion can be detected (Gryspeerdt et al., 2010; Vandekerckhove et al., 2010). The variations in primary replication observed between abortigenic and neurovirulent EHV1 variants may be explained by the differences in induction or susceptibility toward the antiviral effects of IFN.

First, we hypothesized that abortigenic EHV1 strains might be more efficient in down-regulating the synthesis of type I IFN in the epithelium of the URT, in contrast to the neurovirulent strains. In the current study, two abortigenic (97P70 and 94P247) and two neurovirulent (03P37 and 95P105) EHV1 strains were used. When evaluating the viral replication of the different EHV1 strains in mucosal explants and EREC, we observed viral plaques in the epithelium starting from 24 hpi, which increased in number and latitude over time. The observations in the nasal mucosa explants were consistent with previous *in vivo* and *ex vivo* studies (Gryspeerdt et al., 2010; Vandekerckhove et al., 2010). The lateral spread and number of plaques in the epithelium was more pronounced when using the abortigenic variants, while neurovirulent strains induced less and smaller plaques, especially at 48 and 72 hpi. Interestingly, at both time points, we could detect an increased expression of IFNα for all EHV1 variants. An important finding is that abortigenic and neurovirulent EHV1 replication stimulated the secretion of IFN by the mucosal cells, suggesting no obvious interference of either viral variant with the induction of IFN in the URT. Moreover, the 97P70 and 94P247 abortigenic strains could induce the largest viral plaques in the presence of high IFNα concentrations. Both neurovirulent strains reached a replication plateau when IFNα was up-regulated. Similarly, in tracheal mucosa explants, we observed viral plaques starting from 24 hpi. Surprisingly, we observed more and larger viral plaques in the tracheal tissues, compared to the nasal mucosa, despite the presence of more IFNα. The higher number of larger viral plaques in tracheal mucosa explants can possibly be explained by the morphology of the tracheal mucosa. Integrity of the epithelial intercellular junctions (ICJ) progressively decreases from proximal to distal airways, which may benefit the virus to reach its receptor (Lopez-Souza et al., 2009). Indeed, Van Cleemput et al. (2017) recently showed that EHV1 more efficiently binds to tracheal mucosal explants, when compared to nasal septum mucosal explants due to receptor availability. Moreover, only upon a simultaneous destruction of the nasal septum's epithelial integrity and its mucoprotein network, EHV1 could bind efficiently to the explants. In addition, the actin-depolymerizing drug cytochalasin D enhances HSV1 release and spread upon disruption of cell polarity (Schelhaas, 2003). It can be postulated that, due to the different polarity of tracheal and nasal epithelial cells, the lateral spread of EHV1 differs in both tissues, which might explain the reduced strain variant-related differences in the tracheal mucosa. These results indicate that EHV1 replication is strain and tissue specific. In EREC, derived from tracheal mucosa, we could detect IFNα by IF staining, while ELISA or a CPE-reduction assay could not detect IFNα. As described for the mucosal explants, we observed high EHV1 replication in EREC, which might be due to the low IFNα concentration. In comparison with the tracheal mucosa explants, phenotype differences were less pronounced. Overall, the nasal mucosa is the primary site of EHV1 replication, and thus the most important tissue to analyze. This does not necessarily imply an enhanced EHV1-replication in the nasal tissues, but possibly indicates a better adaptation of the virus to specialized anti-viral proteins, such as type I IFN. In the present study, we demonstrated that both EHV1 phenotypes equally induce IFNα in the URT starting from 48 to 72 hpi. This is earlier in infection then observed in previous *in vivo* studies, where the IFN concentration in nasal secretions peaked at day 4 and day 7 pi (Bridges and Edington, 1986; Edington et al., 1989; Gryspeerdt et al., 2010). This can be clarified by the *ex vivo* explant model used in this study, in which the infection dose is much higher compared to the *in vivo* situation. We could observe differences in IFN concentration and bioactivity between mucosae isolated from nasal septum and proximal trachea. In nasal mucosa explants, we observed high concentrations of IFNα by ELISA, while almost no bioactivity could be detected by the CPE reduction assay. IFN not only has beneficial protective effects on the host, but high concentrations or long-term exposure can induce side effects, such as nausea, fever, leukopenia and autoimmune disease (Gutterman, 1994). Therefore, to temper and regulate the immune response not all secreted IFNα may be bioactive in the nasal cavity. This observation has already been verified in many body fluids, such as serum, urine, saliva and peritoneal fluids, in which soluble type I IFN receptors modulate cytokine activity (Hardy et al., 2001). Indeed, a soluble IFNAR can act as an antagonist of the IFN signaling in normal cells (Hardy et al., 2001; de Weerd et al., 2007). However, when incubating rEqIFNα with supernatant of nasal and tracheal mucosa explants, no indications were found for the presence of a soluble IFNAR (data not shown). The underlying mechanism for the low IFNα bioactivity in the nasal mucosa explants therefore remains unclear. Our results suggest that the induction of type I IFN is cell-type and virus strain specific. Indeed, in equine endothelial cells, derived from the pulmonary artery, the neurovirulent T953 EHV1 strain could induce type I IFN at early stages of infection (Sarkar et al., 2015). Later in infection, this neurovirulent EHV1 strain could interfere with IFN transcription and translation. Other researchers have shown that both neurovirulent and abortigenic EHV1-inoculated PBMC could produce equal amounts of IFN (Wagner et al., 2011). A study of Holz et al. (2017) reported differing IFNα levels in nasal secretions and cerebrospinal fluid of horses infected experimentally with the neurovirulent and abortigenic phenotype.

Since the epithelial cells inoculated with abortigenic and neurovirulent EHV1 showed no differences in the bioactive IFNα concentration, we hypothesized that there could be differences in sensitivity between both EHV1 variants upon the antiviral effects of IFNα. To assess the sensitivity of both EHV1 variants to the IFN-induced antiviral state, exogenous rEqIFNα (0, 10, 100, and 1000 U/ml) was added to our *ex vivo* and *in vitro* model, 18 h prior to and during EHV1 inoculation. Here, we show that the replication of both abortigenic and neurovirulent EHV1 could be suppressed by the IFN-induced antiviral responses in the mucosa explants and EREC. Both immediate early and late viral proteins were similarly suppressed in the presence of rEqIFNα (data not shown). As the expression of late viral proteins is indicative of a full viral replication, only these proteins were further used in this study. Strong interferon responses in the URT might aid rapid clearance of the virus, which might explain the mild/subclinical respiratory disease *in vivo* (van Maanen, 2002; Gryspeerdt et al., 2010). Both strain variants showed a reduction in the number of plaques in a rEqIFNα concentration-dependent manner, indicating that IFNα interferes with EHV1 replication in the epithelial cells of the URT. Which steps in viral replication are blocked is not known. Sainz and Halford (2002) observed a similar inhibition of viral replication in Vero cells pretreated with type I IFN during an infection with HSV1. Viral replication was reduced by 20-fold to more than 1,000-fold when cells were pretreated with 100 U/ml of IFNα and IFNβ, respectively. The activation of the IFN-signaling pathway leads to the expression of hundreds of ISG (interferon-stimulated genes), which collectively target several steps of the virus life cycle (Schoggins and Rice, 2011). Several alphaherpesviruses have evolved strategies to evade the IFN response, in order to create time to spread between adjacent cells such as epithelial cells, neurons and fibroblasts across cell junctions (Dingwell and Johnson, 1998; Hukkanen et al., 1999; Johnson et al., 2001). Notably, we found indications for a concentration-dependent difference in plaque latitudes and virus titers between abortigenic and neurovirulent strains. When respiratory epithelium was pretreated with 100 U/ml prior to the abortigenic EHV1 infection, we observed an increased plaque latitude and virus titer, compared to the 10 U/ml. Due to the large biological variation between horses in this study, the increase in plaque latitudes and virus titer was not statistically significant at the concentration of 100 U/ml. Because the enhanced lateral spread of EHV1 was not observed with the neurovirulent variants during treatment with IFNα, this may indicate a variant-specific mechanism to interfere with the antiviral activity of IFN. This would imply that specific ISG-proteins do not suppress, but rather promote the lateral spread of the abortigenic variants at the physiological concentration between 10 and 100 U/ml of rEqIFNα. It is interesting that the indications for a potential pro-viral effect of IFN for the abortigenic strains were observed at a concentration of 100 U/ml, as this concentration corresponds with the IFN bioactivity detected in our *ex vivo* model and in the *in vivo* experiments of Bridges and Edington (1986) and Gryspeerdt et al. (2010). The exact mechanisms of action of the majority of the ISG-encoded proteins are poorly understood, making it difficult to explain a putative pro-viral effect. However, some ISG-effector proteins have been shown before to promote herpesvirus replication, independently of their anti-viral functions. Indeed, Speer et al. (2016) observed a pro-viral effect of ISG15 in human cytomegalovirus (HCMV) and HSV1 infections, by destabilizing the IFNAR-complex the IFN signaling pathway is inhibited. Furthermore, glycoprotein B of HCMV is able to induce viperin, an ISG-protein, which binds to a mitochondrial thiolase to reduce the intracellular ATP concentration. This results in actin disruption and reduced ICJ integrity, which HCMV exploits for cell-to-cell spread (Seo et al., 2011; Schneider et al., 2014). Depending on the prevailing anti-viral or pro-viral effects, viral replication and spread is either suppressed or enhanced. This may help to explain the differences between abortigenic and neurovirulent strains observed in nasal mucosa explants. One speculative explanation could be that the abortigenic strains decrease the ICJ integrity in an IFN-concentration-dependent manner (100 U/ml) in the nasal mucosa via ISG-encoded proteins, while neurovirulent strains may be less efficient in this mechanism. Since the ICJ integrity of tracheal mucosa explants is physiologically reduced compared to that of the nasal mucosa, such an explanation may also fit in our observation of a more efficient lateral spread of both abortigenic and neurovirulent variants in trachea mucosa explants. When tissues are treated with high concentrations of IFN (1000 U/ml), the anti-viral effects rule over any pro-viral effects that IFN may have and inhibit the direct transfer of EHV1 from cell-to-cell. Current research is aimed at further examining the role of ISG in the lateral spread of EHV1.

The indications for differences in IFN-sensitivity between abortigenic and neurovirulent strains were confirmed by the use of an interferon signaling inhibitor, Ruxolitinib (Rux). Rux is a specific inhibitor of the JAK/STAT pathway, inhibiting Janus kinase 1 (JAK1) (Ganetsky, 2013; Assi et al., 2018), thereby impairing the phosphorylation of STAT and further downstream signaling (Stewart et al., 2014; Gage et al., 2016). Increasing the concentration of Rux led to the down-regulation of the IFN-induced antiviral effects without causing cellular cytotoxicity. The most obvious effect of Rux that was noted during the current study was an increase in the typically smaller plaque size of the neurovirulent strains in nasal mucosa explants as demonstrated *ex vivo* and *in vivo* by Vandekerckhove et al. (2010) and Gryspeerdt et al. (2010), respectively. Plaque sizes of the abortigenic strains, which are typically larger than those of neurovirulent strains, were not increased in the presence of Rux. This is in line with the effects of rEqIFNα on plaque size of abortigenic and neurovirulent strains. Indeed, together, these data strongly suggest that plaque formation by the abortigenic strains in nasal mucosa explants is less sensitive to the antiviral effects of IFN compared to neurovirulent strains, suggesting that abortigenic strains have developed additional mechanisms to inhibit/exploit the IFN signaling pathway. In future research it will be of interest to elucidate the molecular mechanisms underlying the different IFN evasion mechanisms of the abortigenic and the neurovirulent EHV1 variants.

Previous research demonstrated many differences between the abortigenic and neurovirulent strain variants, both at the primary site of replication and during cell-associated viremia. It will be interesting to determine the complete sequence of the strains to help identify possible causes of the observed phenotypic differences. During EHV1 infection of the URT, immune cells migrate into the airways to shut down viral replication. During a neurovirulent EHV1-infection of the URT, large amounts of immune cells mobilize into the respiratory mucosa and become infected with EHV1. These cells then may carry the virus to lymphoid tissues and the blood circulation, to reach the central nervous system. This is an explosive and precarious mechanism particularly displayed by neurovirulent variants, and in most of the infections, results in the elimination of the virus because of the massive mobilization of immune cells. However, the abortigenic strains recruit fewer leukocytes into the URT, which enables the virus to replicate in the respiratory epithelium to promote viral shedding. Occupant immune cells become infected with abortigenic EHV1 in a less explosive and more controlled manner, followed by a silent transport in these “Trojan horses” to the pregnant uterus (Vandekerckhove et al., 2010; Laval et al., 2015; Zhao et al., 2017). In general, our findings support the speculation that the abortigenic variants may have a selective advantage over the neurovirulent variants for long-term maintenance within the equine population, by mastering the antiviral effect of IFN at the URT (Nugent et al., 2006; Goodman et al., 2007; Vandekerckhove et al., 2010; Laval et al., 2015; Zhao et al., 2017).

In summary, the present study is the first to provide a better understanding of the innate IFN response upon abortigenic and neurovirulent EHV1 infection at the respiratory mucosa surface. Using the *ex vivo* mucosa explant model, we were able to detect strong IFNα responses upon abortigenic and neurovirulent EHV1 infection. The enhanced replication of abortigenic EHV1, compared to replication of neurovirulent EHV1, at the physiological IFNα concentration, suggests that EHV1 abortigenic strains interfere more efficiently with innate immune responses of the host in the respiratory mucosae. In conclusion, an effective IFN response protects the horse to a moderate (abortigenic strains) or high (neurovirulent strains) extent against an EHV1 infection of the respiratory tract.

## Ethics statement

This study was carried out in accordance with the recommendations of The Belgian and European legislation, EU directive 2010/63/EU. The protocol was approved by the Ethical Committee of the Faculties of Veterinary Medicine and Bioscience Engineering, Ghent.

## Author contributions

KP designed and performed all the experiments, statistically evaluated the results, designed the figures, and wrote the first draft of the manuscript. JV helped to perform experiments. KL helped to design the experiments. GS and RM provided the protocols for primary equine respiratory epithelial cell isolation and cultivation. HF and HN are the promoters of KP and designed the experiments. All authors reviewed the manuscript.

### Conflict of interest statement

The authors declare that the research was conducted in the absence of any commercial or financial relationships that could be construed as a potential conflict of interest.
