# Supplementary material for: Abortigenic but Not Neurotropic Equine Herpes Virus 1 Modulates the Interferon Antiviral Defense
Source: Front Cell Infect Microbiol. 2018 Sep 12;8:312. doi: 10.3389/fcimb.2018.00312 (PMC6144955; doi:10.3389/fcimb.2018.00312)
Supplement: Supplementary file 1 [file Data_Sheet_1.pdf]

Legends supplementary figures

Supplementary Figure 1: *Evolution of plaque formation in mucosa explants from the nasal septum, trachea and primary respiratory epithelial cells (EREC).* Respiratory mucosa explants and EREC were inoculated with two abortigenic (97P70 and 94P247) and two neurovirulent (03P37 and 95P105) EHV1 variants. At 10, 24, 48 and 72 hpi, explants and supernatants were collected and analyzed. Fifty cryosections were made and immunofluorescence staining was performed, to visualize EHV1-induced plaques. (**A**) The number of viral plaques and (**B**) plaque sizes were determined and statistically analyzed for each EHV-1 strain and compared between the included strains. (**C**) Virus titers in supernatants, of each viral variant, were obtained by collecting supernatant from mucosa explants and EREC. Different letters represent significant differences ($p < 0.05$) in the number of viral plaques. Experiments were performed on mucosa explants of 3 horses (1 ●, 2 ■ and 3 ▲) and EREC of 3 other horses (I ○, II □ and III △).

Supplementary Table 1: *The occurrence of apoptosis in the epithelium and lamina propria in equine respiratory epithelial cells 18 h pretreated and cultured with 0, 10, 100 or 1000 U/ml rEqIFNα during 48 h.* No significant increase of apoptosis was observed in the epithelium and the lamina propria of mucosa explants. The maximum percentage of TUNEL-positive cells in the epithelium and lamina propria was 2.2 ± 2.9 % and 3.2 ± 1.6 %, respectively.

Supplementary Figure 2: *The effects of rEqIFNα on EHV1 inoculum.* EHV1 inoculum prepared in medium containing 10, 100 or 1000 U/ml rEqIFNα was titrated on RK-13 cells, and compared to inoculum prepared in rEqIFNα-negative medium. No significant differences were observed. All data are expressed as the mean value of three experiments ± standard deviation.

Supplementary Table 2: *The effects of Rux on EHV1 inoculum.* EHV1 inoculum prepared in medium containing 4 μM Rux was titrated on RK-13 cells, and compared to inoculum prepared in equivalent concentrations of DMSO. No significant differences were observed. All data are expressed as the mean value of three experiments ± standard deviation.

Supplementary Figure 3**:** *Occurrence of apoptosis in the epithelium and lamina propria in Rux-treated equine respiratory epithelial cells.* This table represents the effects of 2 h pretreatment and cultivation with Rux or equivalent DMSO of mucosa explants until 48 h. No significant increase of apoptosis was observed in the epithelium and the lamina propria in equine explants of the deep intranasal part of the septum and the trachea.
