# Supplementary figures and images for: Abortigenic but Not Neurotropic Equine Herpes Virus 1 Modulates the Interferon Antiviral Defense
Source: Front Cell Infect Microbiol. 2018 Sep 12;8:312. doi: 10.3389/fcimb.2018.00312 (PMC6144955; doi:10.3389/fcimb.2018.00312)

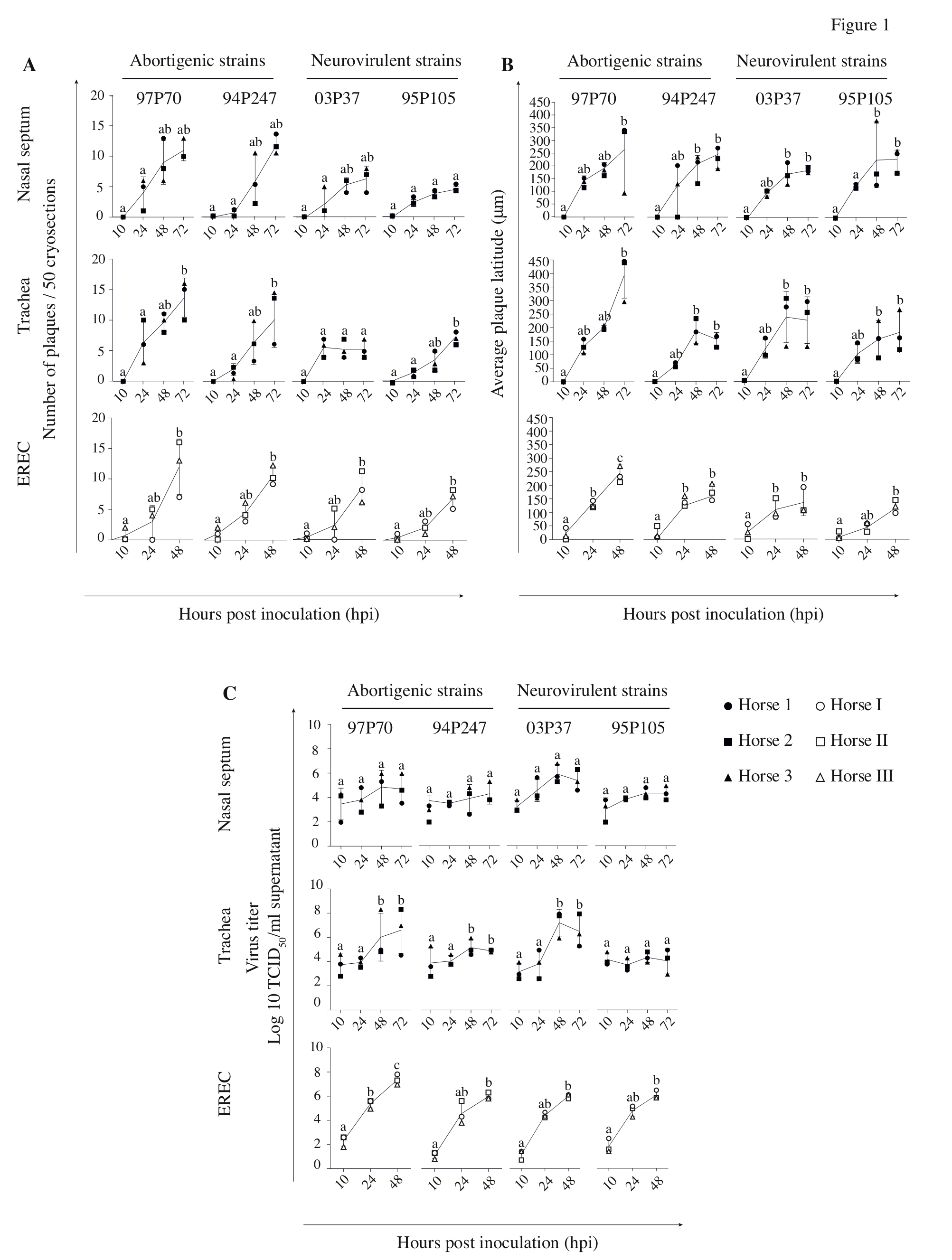

Supplement: Supplementary file 2 [file Image_1.TIF]

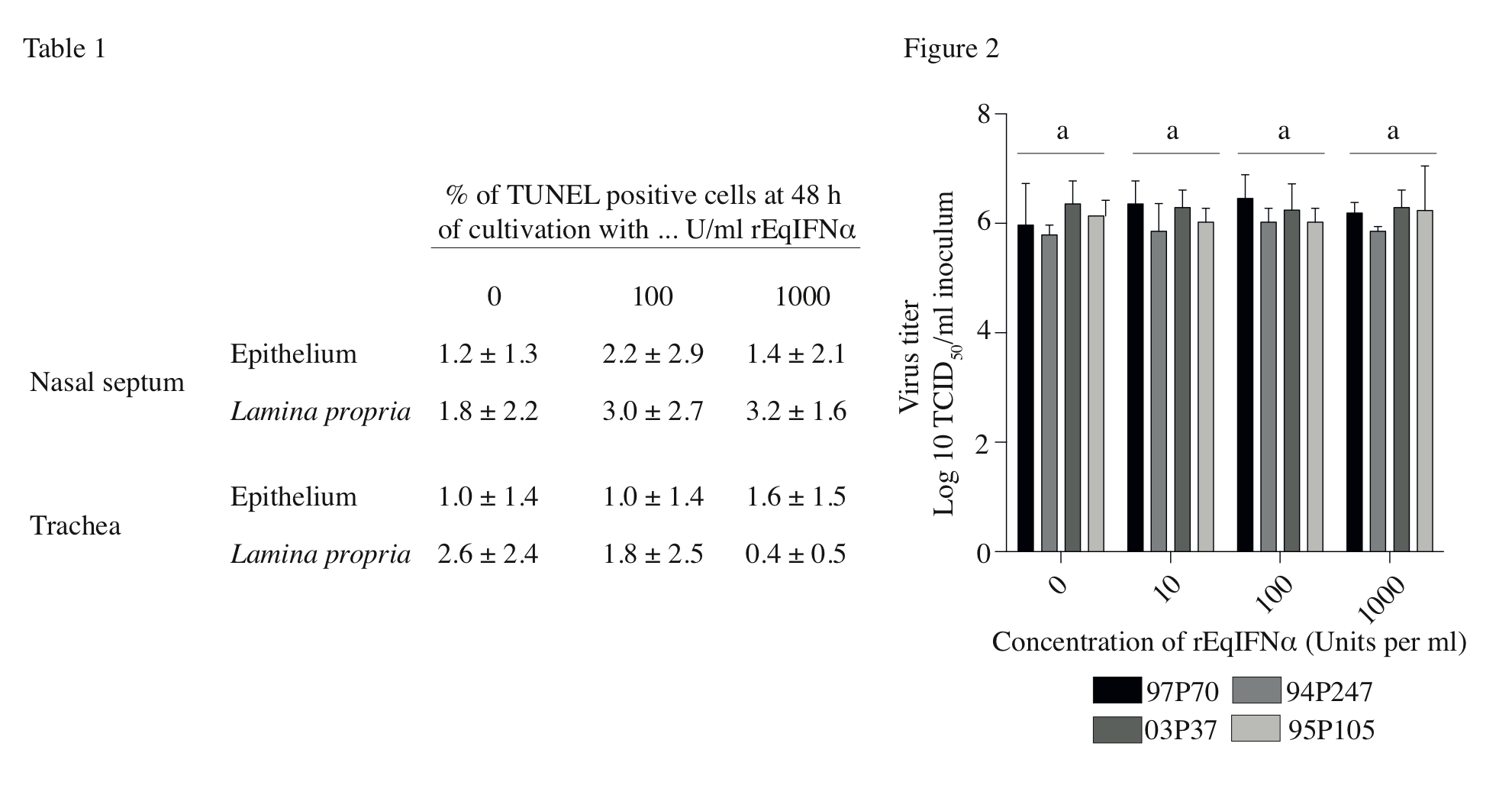

Supplement: Supplementary file 3 [file Image_2.TIF]

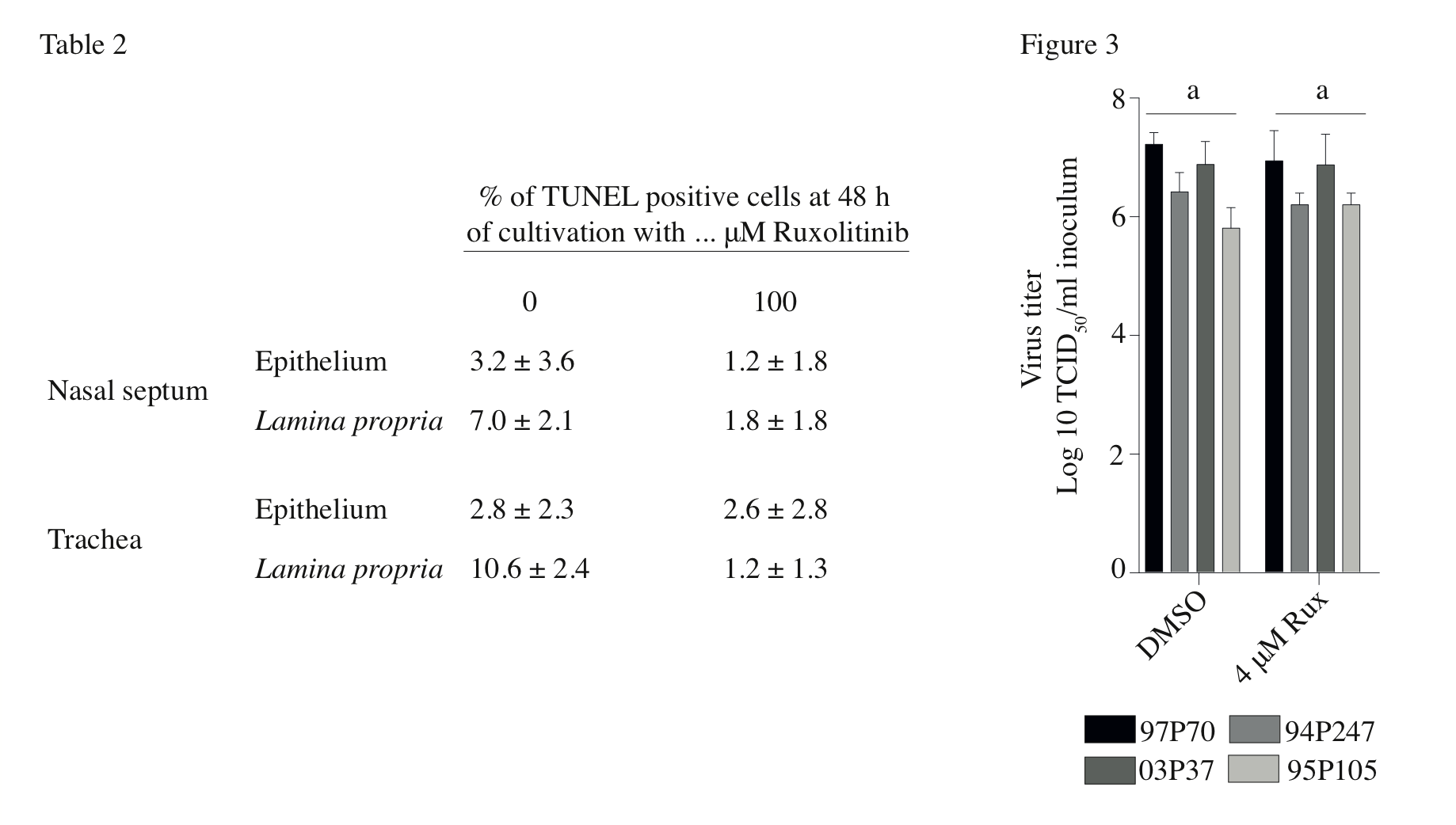

Supplement: Supplementary file 4 [file Image_3.TIF]
